# Calcium Ion Flow Permeates Cells through SOCs to Promote Cathode-Directed Galvanotaxis

**DOI:** 10.1371/journal.pone.0139865

**Published:** 2015-10-08

**Authors:** Liang Guo, Chunyan Xu, Dong Li, Xiulan Zheng, Jiebing Tang, Jingyi Bu, Hui Sun, Zhengkai Yang, Wenjing Sun, Xiaoguang Yu

**Affiliations:** 1 Department of Biochemistry and Molecular Biology, College of Basic Medical Science, Harbin Medical University, Harbin, Heilongjiang 150081, P.R. China; 2 Institute of Biomedical Engineering, College of Automation, Harbin Engineering University, Harbin, Heilongjiang 150001, P.R. China; 3 Bioinformatics Research Center, College of Automation, Harbin Engineering University, Harbin, Heilongjiang, 150001, P.R. China; 4 Department of Ultrasonography, Harbin Medical University Cancer Hospital, Harbin, Heilongjiang 150081, P.R. China; 5 Department of Clinical Oncology, Harbin Medical University Cancer Hospital, Harbin, Heilongjiang 150081, P.R. China; Cinvestav-IPN, MEXICO

## Abstract

Sensing and responding to endogenous electrical fields are important abilities for cells engaged in processes such as embryogenesis, regeneration and wound healing. Many types of cultured cells have been induced to migrate directionally within electrical fields in vitro using a process known as galvanotaxis. The underlying mechanism by which cells sense electrical fields is unknown. In this study, we assembled a polydimethylsiloxane (PDMS) galvanotaxis system and found that mouse fibroblasts and human prostate cancer PC3 cells migrated to the cathode. By comparing the effects of a pulsed direct current, a constant direct current and an anion-exchange membrane on the directed migration of mouse fibroblasts, we found that these cells responded to the ionic flow in the electrical fields. Taken together, the observed effects of the calcium content of the medium, the function of the store-operated calcium channels (SOCs) and the intracellular calcium content on galvanotaxis indicated that calcium ionic flow from the anode to the cathode within the culture medium permeated the cells through SOCs at the drift velocity, promoting migration toward the cathode. The RTK-PI3K pathway was involved in this process, but the ROCK and MAPK pathways were not. PC3 cells and mouse fibroblasts utilized the same mechanism of galvanotaxis. Together, these results indicated that the signaling pathway responsible for cathode-directed cellular galvanotaxis involved calcium ionic flow from the anode to the cathode within the culture medium, which permeated the cells through SOCs, causing cytoskeletal reorganization via PI3K signaling.

## Introduction

Endogenous direct-current electrical fields (EFs) are present in many organisms and play significant roles in a number of physiological processes, including embryonic development, regeneration, wound healing, and tumor invasion and metastasis [1, 2]. The electrical fields in intact *Xenopus* embryos are generated by spatial differences in the transepithelial potentials, with currents exiting the blastopore at densities as high as 100μA/cm^2^. The sites where such currents exit the embryo are major regions of tissue reorganization, and disrupting the normal electrical current in an embryo can lead to developmental defects. In adult organisms, disruption of epithelial integrity due to injury causes the electrical fields to be oriented toward the wound (0.4–1.4 V/cm), and the directional migration of the surrounding epithelial cells can be disrupted by interfering with the electrical fields [3].

In vitro, the application of EFs at physiological strength induces many different types of cells to respond with directed migration [4]. These cells can move with directional preference toward the cathode or anode in electrical fields. Most cells migrate toward the cathode [5, 6], whereas a minority of cells migrate toward the anode [7, 8].

The distributions of membrane components and intracellular organelles and the intracellular signaling pathways that are activated by EFs have only recently been clearly identified [9–13]. But to precisely understand the mechanism by which cells sense the EFs and transduce them into intracellular signals remain controversial. Although some in vitro experiments had demonstrated that membrane components were redistributed in EFs [14–19], it has remained difficult to explain how cells within tissues redistribute membrane components in vivo because these cells are adhered to one another in three dimensions. Therefore, the redistribution of membrane components is more likely to be the second step in EF-signal transduction, followed by the intracellular distribution of many organelles, although some changes can occur rapidly. For example, epidermal growth factor receptors can redistribute in as little as 10 min after the onset of an EF in vitro [10]. In addition, it is difficult for intracellular organelles to undertake the primary function of sensing EFs during migration.

It has commonly been observed that currents produced by wound-mediated EFs and those occurring during development resulted from the directional flow of charged ion species that were present in the cytoplasm and extracellular fluid (e.g., Na^+^, Cl^-^, K^+^, and Ca^2+^) [3, 20] and that these EFs were likely to be the original signals sensed by the cells. In this study, we selected the access point of ionic flow to identify the cellular sensor of external electrical fields.

In this paper, we present a new electrotaxis system that utilizes polydimethylsiloxane (PDMS) with which cell migration toward the cathode or anode was investigated using pulsed direct current EF, constant direct current EF and other methods. We found that calcium that passed through cells at the drift velocity from the anode to the cathode may have promoted cathode-oriented cell migration.

## Materials and Methods

### Cell Culture

This study was carried out in strict accordance with the recommendations in the Guide for the Regulations for the Administration of Affairs Concerning Experimental Animals, and was approved by the animal care and welfare committee of Harbin Medical University. Surgeries were performed under sodium pentobarbital anesthesia, and all efforts were made to minimize suffering.

For fibroblast isolation, postnatal day 1 mice were anesthetized. Dorsal skin was collected under sterile conditions and digested in 0.25% collagenase A in PBS for 20 min in a 37°C shaking water bath. Cells were then pelleted by centrifugation and resuspended in growth medium, consisting of DMEM, 10% FBS, and 1% penicillin/streptomycin. In these experiments, cells were used at passages 3 to 10. Human prostate cancer PC3 cells were obtained from the American Type Culture Collection (ATCC, Manassas, VA, US).

### Device Fabrication and Operation

**Fig 1:**
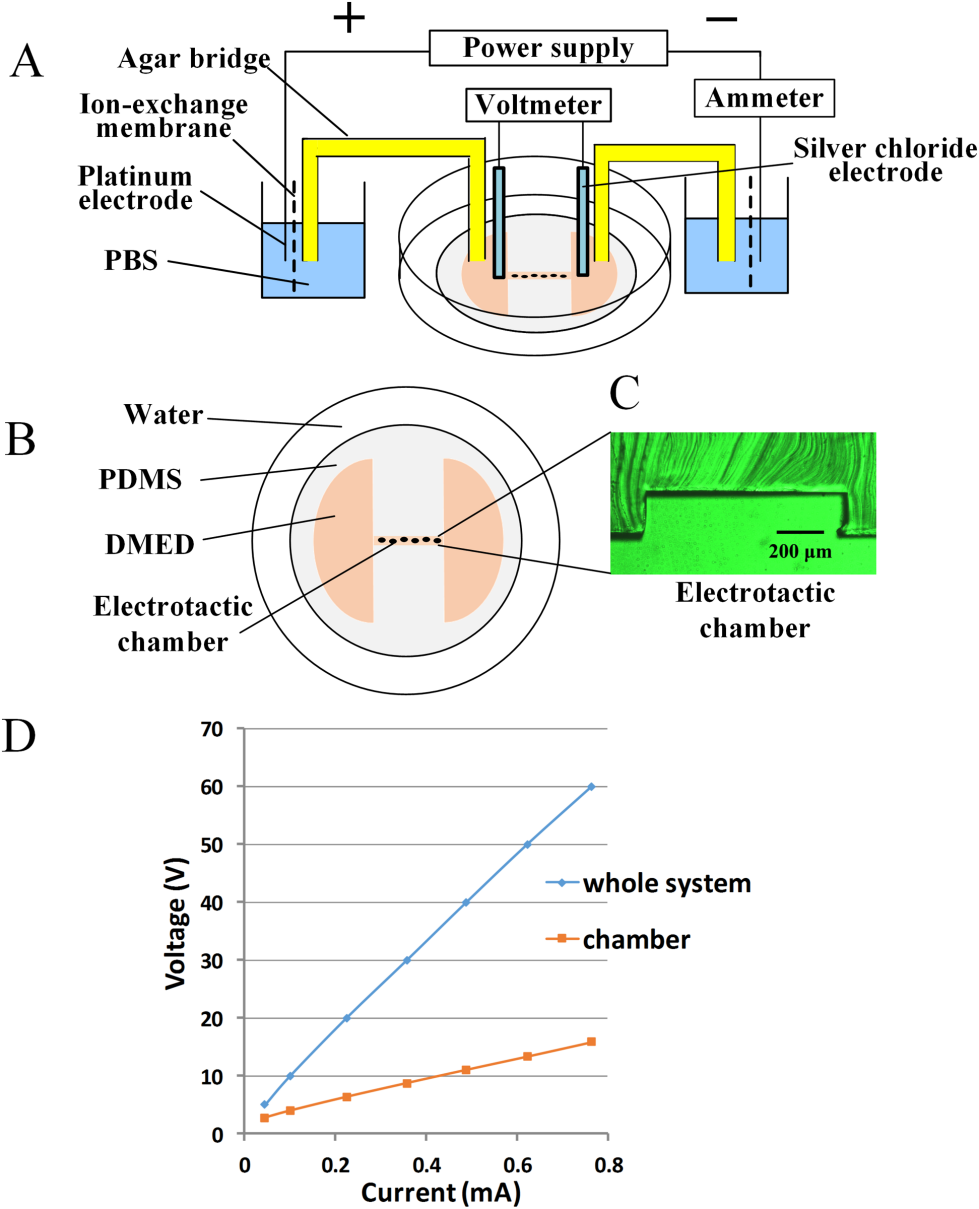
System for Electrotaxis Experiments. (A) Schematic drawing of the electric field application. (B) The galvanotaxis chamber was constructed with PDMS in a Petri dish, shown from above or (C) as a transverse section. (D) The total voltage applied to the system and the voltage on the galvanotaxis chamber changed with the current.

A glass strip (20 mm×0.8 mm×0.17 mm) was attached to the base of a polystyrene Petri dish (60 mm×15 mm) as the mold to shape a PDMS galvanotaxis chamber (20 mm×0.8 mm×0.17 mm). PDMS prepolymer (DOW CORNING, Midland, Michigan, US) was mixed with curing reagent at a 10:1 mass ratio and spread onto the base of the Petri dish to achieve a thickness of 1 mm. PDMS was then cured by heating in an oven. A PDMS layer was obtained by peeling it off from the Petri dish. Then, two semicircular holes (approximately 0.5 cm^3^) were cut in the PDMS layer as wells to retain the medium to which the strip chamber was connected. The PDMS layer was sterilized using ultraviolet light for 1 h per face and adhered to a glass Petri dish (90 cm) cleaned with sulfuric acid. Water was placed around the PDMS layer in the dish to maintain humidity. The cells were attached to the chamber one day prior to the experiment. The dish was covered with a specially prepared lid containing two holes with agar bridges running across the holes. A constant direct current electrical field was provided by a direct current power supply, and a pulsed direct current was provided by a signal generator. The currents were applied to cells through two platinum electrodes immersed in PBS-filled reservoirs that were connected to the wells at each end of the galvanotaxis chamber by two 6-cm-long agar bridges (3% agar in PBS) (Fig 1A–1C). The current through the cells was monitored continuously during the experiment with an ammeter in series. An anion-exchange membrane (Lvhe, Hangzhou, Zhejiang, China) was attached between an agar bridge and a platinum electrode to produce anionic flow when necessary. The chamber was placed on a digital microscope (MOTIC AE31 Xiamen, Fujian, China) stage in an incubator with 5% CO_2_ at 37°C and recorded. We tested the galvanotaxis system for more than 20 hours, and the cells grew well (S1 Movie).

### Electrical Field Measurements

Prior to beginning the cellular experiments, we measured the resistance of the electrode and the agar bridges. Silver chloride electrodes were integrated into the two wells that contained the medium. Using the ammeter, we recorded the voltage in the chambers or the bridges as different electrical currents were applied. There was a linear relationship between the voltage and the current in the entire galvanotaxis system as well as in the small chamber (Fig 1D). Therefore, for a cellular galvanotaxis experiment, the silver chloride electrodes could be removed. Thus, we could modulate the voltage of the galvanotaxis system to apply electrical fields of any intensity (0.75–8 V/cm) to the cells in the chamber by measuring the current with an ammeter, which simplified the system.

### Quantification of Cellular Behavior

Cell migration was recorded using time-lapse microscopy at a rate of 1 frame/ 6 min for 7 h. The movement was studied using Motic Images Plus 2.0 ML software (Motic, Xiamen, Fujian, China). The position of a cell in each frame was determined from the position of the nucleus, which was manually identified.

The mean speed was defined as the total displacement from the starting position of each cell to its new location, divided by the total experimental period.

The vector speed was defined as the displacement in the direction of the EF vector divided by the total experimental period.

The directionality of cellular translocation was expressed as cos θ and was calculated from the angle created by the translocation vector of each cell with respect to the direction of the imposed electrical fields, using the following formula: average cosθ = Σ_i_cosθ/n. Data were collected from three individual experiments.

### [Ca2+] Imaging

[Ca^2+^]_i_: Fluo-4FF AM (Life-Technologies, Eugene, Oregon, US) was used to record the intracellular calcium levels. The cells were loaded using 5 μM Fluo-4FF AM at 37°C for 45–60 minutes. After washing, the cells were incubated for an additional 30 minutes and were then examined using an inverted microscope (Olympus IX71). A 100 W mercury lamp and a 450–490 nm filter were used for excitation; the emission was recorded using a 495-nm long-pass beam splitter and a 500–550 nm emission filter. The exposure time was 10 minutes, and the sampling rate was 15 seconds between frames; alternatively, for measurements of longer time scales, the shutter was open for 30 sec every 5 min. The data are presented as the ratio (F: F0) of fluorescence intensity at a defined time point (F) divided by the intensity immediately prior to the onset of an EF (F0).

[Ca^2+^]_o_: The concentration of Ca^2+^ in the culture medium was determined using the QuantiChrom™ Calcium Assay Kit purchased from BioAssay Systems (Hayward, CA, US).

### siRNA-mediated Knockdown of Orai1 Expression

PC3 cells were transiently transfected with a small interfering RNA (siRNA) (RiboBio Co., Ltd., Guangzhou, China) directed against Orai1 or with negative control siRNA, using Lipofectamine 2000 reagent (Life Technologies, Carlsbad, CA, USA) according to the manufacturer's instructions.

The sequence of the si-h-Orai1 was as follows:

Sense: 5′-CCUUCGGCCUGAUCUUUAU dTdT -3′

Antisense: 3′-dTdT GGAAGCCGGACUAGAAAUA-5′.

At 24 h prior to the transfection, the cells were plated in a 6-well plate. The medium was changed 24 h after transfection and after 48 h of knockdown treatment, the cells were trypsinized and then seeded in the galvanotaxis chamber. The efficiency of silencing was evaluated according to the relevant protein level and the effect of that protein level on galvanotaxis at 72 h.

### Statistics

All of the data are expressed as the mean ± standard error. Statistical significance was determined using an unpaired Student's t-test or one-way ANOVA with a post hoc LSD multiple comparison test. A P value of <0.05 was considered significant.

## Results

### Galvanotaxis of Mouse Fibroblasts and PC3 Cell Lines

Many types of cells respond to EF with cathode-directed migration, although a few types of cells migrate toward the anode [5–7]. In this study, in the absence of an electrical field mouse fibroblasts and PC3 cells migrated in a random, non-directional manner. However, when a 5 V/cm electrical field was applied, the mouse fibroblasts and PC3 cells were strongly inclined to migrate in the direction of the cathode (S1 and S2 Movies). The mouse fibroblasts oriented themselves perpendicular to the field vector (S1 Movie).

To demonstrate our findings, we will first present the results of research on the cathode-directed migration of mouse fibroblasts in detail and then present the results of research on PC3 cells, which utilize the same mechanism used by mouse fibroblasts to move under EF conditions.

### Mouse Fibroblasts Sense the Flow of Ions

**Fig 2:**
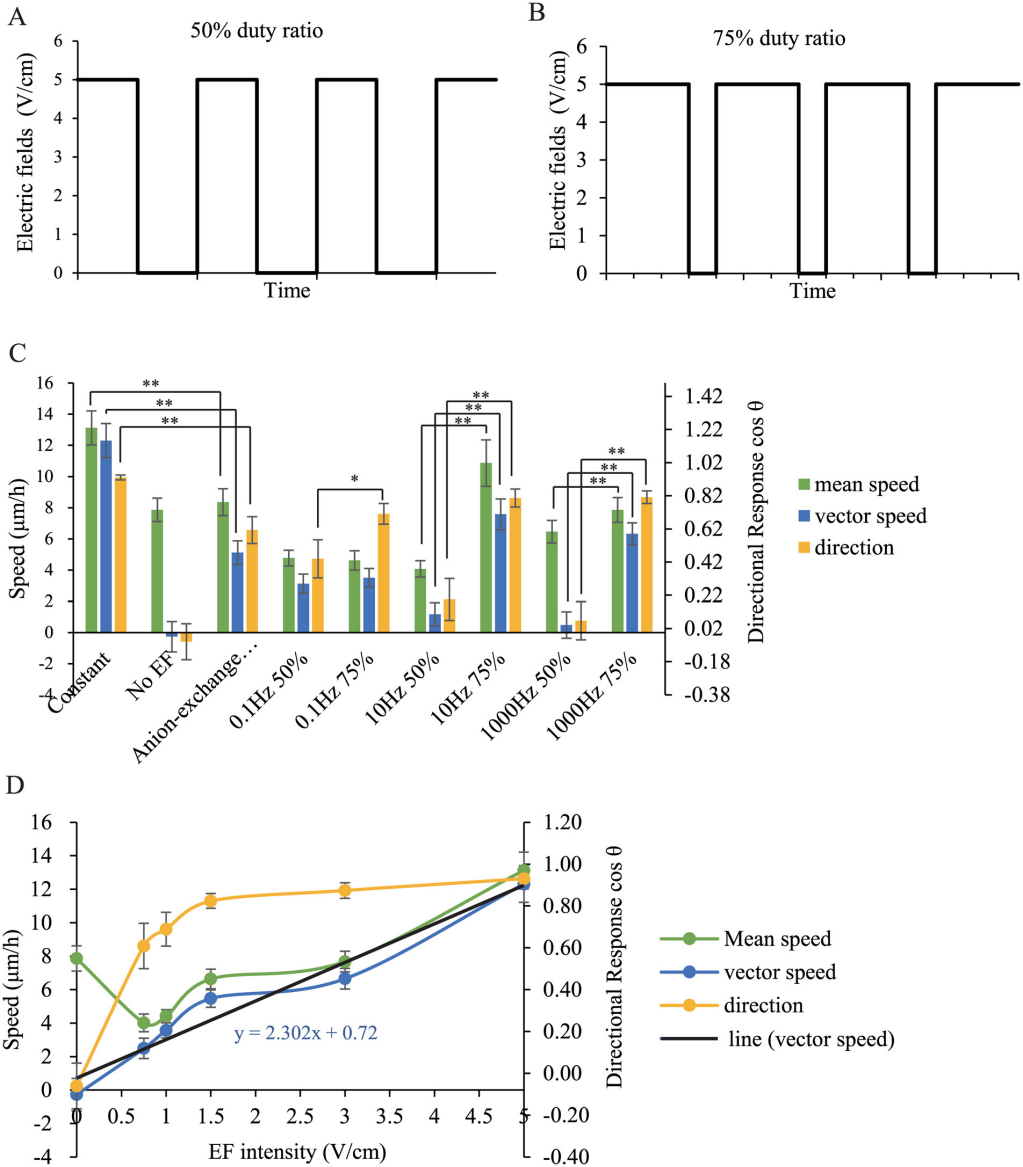
Cell Migration in Pulsed Directional Current Fields, Constant Electric Fields of Decreasing Intensity or an Anion-exchange Membrane Between an Agar Bridge and a Platinum Electrode. Mouse fibroblasts attached to the galvanotaxis chamber were exposed to a direct current electric field for 7 h, and cells’ migration was imaged every 6 min during the experiment. (A) and (B) are pulse direction waveforms of 50% and 75% duty cycles in a 5 V/cm field. (C) Mean speed, vector speed and average cosine of the cellular translocation under different pulse EFs. (D) Mean speed, vector speed and average cosine of the cellular translocation with decreasing EF intensity. The linear relationship between vector speed and electric field intensity is y = 1.865x+0.5913. * (p < 0.05) and ** (p < 0.01). Error bars indicate standard error.

To test whether the mouse fibroblasts reacted to the flow of ions or to the difference between the electrical potentials at the ends of the cell, two types of EF were applied. First, the response of the mouse fibroblasts to direct pulse currents was explored. While maintaining a 5 V/cm electrical field, different low-frequency direct pulse currents were applied, including 0.1 Hz, 10 Hz, and 1000 Hz direct pulse currents at a 50% or 75% duty cycle (Fig 2A and 2B). Decreasing the duty cycle from 75% to 50% significantly decreased the directional migration of the cells toward the cathode (Fig 2C). Thus, we concluded that the persistence time of the EF played a major role in galvanotaxis. These results suggested that cells reacted to the flow of ions rather than to the electrical potential at opposite ends of a cell.

Next, we decreased the intensity of the constant electrical field to 5 V/cm, 3/V cm, 1.5 V/cm, 1 V/ cm and 0.75 V/cm and observed the simultaneous decrease in the directional migration of mouse fibroblasts toward the cathode (Fig 2D). We found that there was a linear relationship between the vector speed (in μm/h) and the electrical field intensity (in V/cm), which could be expressed as y = 2.302x + 0.72 (Fig 2D). A direct current field was generated by ion flow in the liquid medium, and the electrical mobility of the ion flow in the EF was not affected by the intensity of the EF. Using the same conductor, the amount of ions that flowed past a point per second at 5 V/cm was five times that at 1 V/cm, but the electrical mobility of the ions was the same. The linear relationship between the vector speed and the electrical field intensity indicated that cell motility was driven by the passage of ions and that the number of ions that passed determined the speed of cell motility.

To confirm this conclusion, we placed an anion-exchange membrane in front of the two electrodes to produce anionic flow alone, while the electrical potentials remained intact. However, the directed migration of mouse fibroblasts under the constant electrical field of 5 V/cm was inhibited by the anion-exchange membrane (Fig 2C).

We concluded that mouse fibroblasts sensed the flow of ions. The amount of ions passing the cells exerted a force on the cells. It remained unclear which ion produced the force, i.e., a cation or an anion, calcium, chlorine or another ionic species.

### Mouse Fibroblast Migration Depends on: the Flow of Calcium Ions

Calcium is associated with cytoskeletal remodeling, myosin light-chain kinase function and the direction of cellular movement [21–27]. Calcium signaling has long been proposed to play a role in the galvanotactic response, whereas sodium, hydrogen, or chloride ions have been excluded from having such a role [28, 29]. Under calcium-free conditions, *Dictyostelium* cells and osteoblasts cease the directional migration and elevation of the intracellular calcium content that was induced by EF [30, 31]. Given these reports, we limited our exploration of ionic flow to calcium ions.

**Fig 3:**
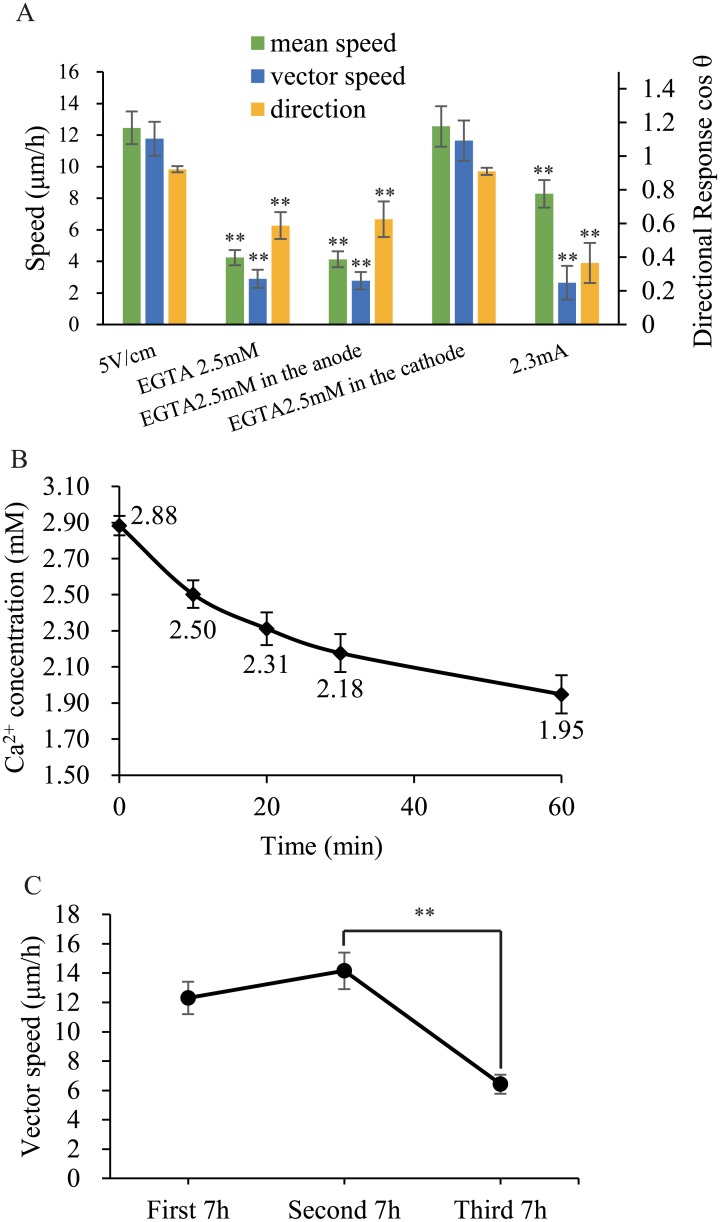
Function of Calcium Ions in Cell Migration in Response to an EF. (A) EGTA (2.5 mM) in only the anode culture medium significantly inhibited mouse fibroblasts’ migration toward the cathode, but EGTA (2.5 mM) in the cathode medium did not inhibit migration. Amplifying the current from 0.115 mA to 2.3 mA abolished the migration in a larger chamber with a cross-sectional area 20 times larger than the original chamber. (B) The concentration of calcium in the anode culture media decreased in the larger chamber over time. (C) Migration speed in the vector direction decreased with time. * (p < 0.05) and ** (p < 0.01) vs EF 5 V/cm control. Error bars indicate standard error.

First, we tested the ability of the calcium chelator EGTA (ethylene glycol tetraacetic acid) (2.5 mM) to restrict the galvanotactic response of mouse fibroblasts and found an obviously restricted function with an EF of 5 V/cm (Fig 3A). Then, to ensure that calcium-ion flow promoted cell migration, 2.5 mM EGTA was added only to the anode medium. Because of the capillary shape of the galvanotaxis chamber (20 mm×0.8 mm×0.17 mm), the effect of the short-term diffusion of EGTA could be ignored. At the beginning of the 2-h time period, the cells demonstrated the same migratory behavior as observed using double-polar EGTA restriction. Next, we exchanged the electrodes, resulting in EGTA being present in the cathode medium. The cells quickly responded to the EF and exhibited no obvious difference in speed or direction of movement compared with when no EGTA was applied (S3 Movie). These experiments confirmed that in an EF, calcium ions are transferred from the anode to the cathode to direct cell motility.

To verify this hypothesis, we measured the change in the calcium ion concentration in the medium and the speed of cell migration in a 5 V/cm EF. To observe this change, we increased the electrical current from 0.11 mA to 2.3 mA by using a larger chamber (20 mm×3 mm×1 mm) with a cross-sectional area 20 times larger than the original chamber, and we shortened the experimental period by 95%. The mean vector speed decreased to 2.3 μm/h over 7 hours, indicating that the cells had not moved (Fig 3A). Next, we measured the calcium concentration of the anode culture medium. In a 0.5 ml sample of anode medium, the calcium concentration was 2.88 mM, and the concentration of calcium ions decreased with time (Fig 3B). This meant that, according to our measurements and calculations, in the presence of a 2.3 mA electrical current, 0.02392 μmol of electrons flowed per second and in the first 30 min, 0.196 nmol of calcium ions flowed from the anode side to the cathode side per second. However, the result indicated that, during the first 30 min, the calcium-ion flow rate was 1.7% that of the total ionic flow rate, whereas during the second 30 min, the calcium-ion flow rate was 0.05% that of the total ionic flow rate, which included H^+^, OH^-^, K^-^, Na^+^, Cl^-^, PO_4_^3-^, SO_4_^2-^ and other ionic species. Thus, due to the decreasing calcium concentration, the calcium ion flow comprised a decreasing proportion of the total ion flow, which caused cells not to move in a 2.3 mA electrical field. This phenomenon should cause a decrease in the speed of cell migration over a relatively long period. To confirm this assumption, cell movement in a 5 V/cm EF at a current of 0.11 mA was analyzed over 21 h and was divided into three phrases to explore the changes in speed. The results indicated that the speed of cell migration decreased after 14 h (Fig 3C).

Taken together, these results demonstrated that calcium ion flow promoted the migration of mouse fibroblasts toward the cathode.

### Mouse Fibroblasts Sense Calcium Ion Flow via Calcium Channels but not via Increased Intracellular Calcium

**Fig 4:**
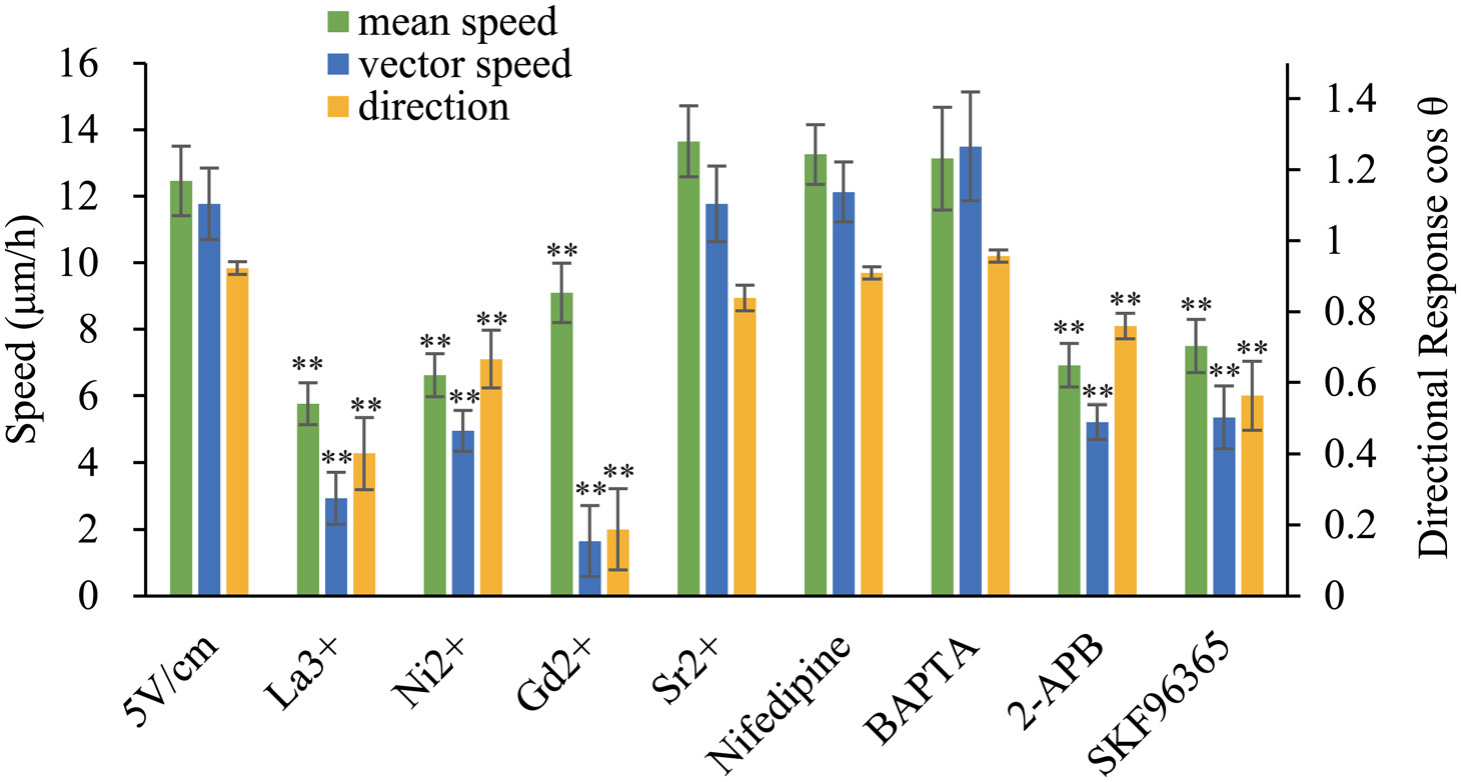
Calcium Channel Blockers Inhibit Cathode-Directed Migration. Several inorganic calcium channel blockers, including La^3+^ (200 μM), Gd^2+^ (100 μM), and Ni^2+^ (300 μM), and store-operated calcium channel (SOC) blockers, including 2-APB (100 μM) and SKF96365 (10 μM), inhibit cathode-directed cell migration, but Sr^2+^ (1 mM), a substitute for calcium, nifedipine (20 μM), a voltage-dependent channel inhibitor, and BAPTA-AM (25 μM), an intracellular calcium chelator, failed to inhibit cellular responses to EF. * (p < 0.05) and ** (p < 0.01) vs EF 5 V/cm control. Error bars indicate standard error.

We next addressed the question of how mouse fibroblasts sense the flow of calcium. Calcium ions comprise only a small proportion of the flowing ions; thus, calcium may target a movement-related intracellular pathway through a special channel in the membrane to circumvent this problem. Ca^2+^channel blockers have been shown to inhibit galvanotaxis in human keratinocytes [32]. The inorganic Ca^2+^ channel blockers gadolinium chloride (100 μM), nickel chloride (300 μM), and lanthanum chloride (200 μM) were applied to examine this possibility. Sr^2+^ was substituted for Ca^2+^ and was applied as a supplement (Fig 4). The results showed that La^3+^, Gd^2+^ and Ni^2+^ inhibited cell migration to the cathode, but Sr^2+^ (100 μM) did not. Moreover, La^3+^, Gd^2+^ and Ni^2+^ did not inhibit the mean speed of the cells (Fig 4 and S4 Movie). We inferred that the cells were unable to sense the direction of EF when the calcium channels were blocked and therefore moved randomly. Next, we tested whether BAPTA-AM, an intracellular-calcium chelator would inhibit the cellular galvanotactic response. However, BAPTA-AM (25 μM) failed to inhibit the cellular response to if it has not (Fig 4), which was consistent with the results of other studies [28, 33]. There were three possible reasons for these results. First, it is possible that calcium did not enter the cells; however, if this was the case, it is unclear how Ca^2+^ channel blockers could inhibit galvanotaxis. Second, perhaps the low (micromolar) concentration of BAPTA-AM in the cells was insufficient to chelate the continuous influx of calcium. Third, perhaps the calcium passed completely through the cells. We examined the third possibility in the subsequent experiments.

### Mouse Fibroblasts: SOCs Mediated the Influx of Calcium Ions

Calcium entry into cells can be accomplished when Ca^2+^-permeable ion channels, including voltage-dependent calcium channels, ligand-gated calcium channels, and store-operated calcium channels (SOCs) open. Mouse epithelial fibroblasts are non-excitable cells that may not express voltage-dependent calcium channels and may express ligand-gated calcium channels at low levels. However, SOCs may be the major route for calcium influx into these cells. It has been reported that many eukaryotic cells generate spatially and temporally encoded intracellular signals through extracellular calcium entry via SOCs [34–36]. We used the SOC antagonists SKF96365 (10 μM) and 2-aminoethoxydiphenyl borate (2-APB) (100 μM) to evaluate the effect of SOCs on the mouse epithelial fibroblast migration that was induced by EF using nifedipine, an L-type calcium-channel blocker, as a control. The results (Fig 4 and S5 Movie) showed that SKF96365 and 2-APB significantly inhibited directional cell movement toward the cathode; however, nifedipine (25 μM) did not.

These results are consistent with the hypothesis that SOCs mediated the calcium ion influx induced by EFs.

### Mouse Fibroblasts Show No Increased or Asymmetrical Calcium Distribution in an EF

**Fig 5:**
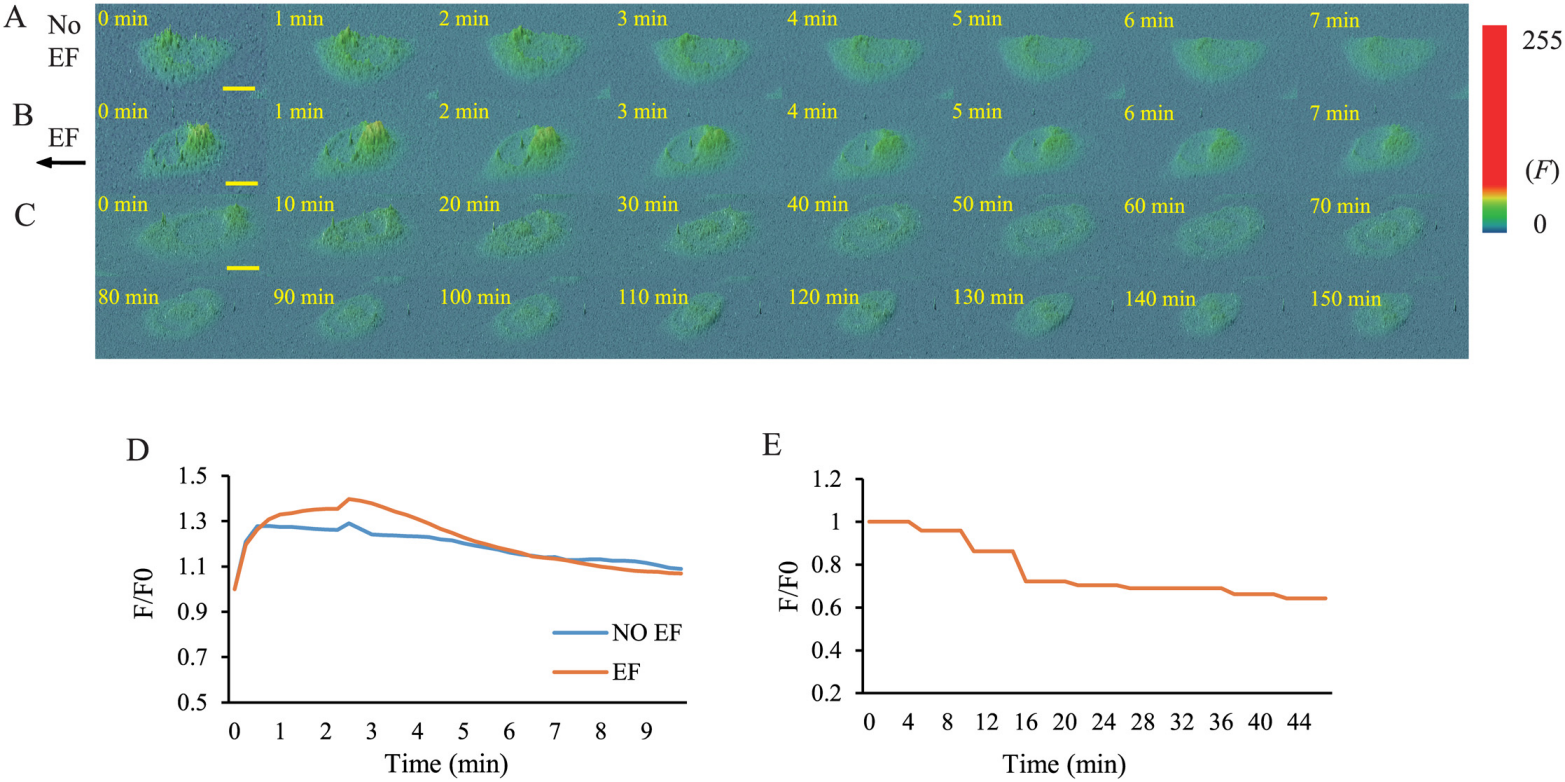
Calcium Distributions and Concentrations In Control Cells and Cells Exposed to EFs. (A) Calcium distributions in mouse fibroblasts without EFs as imaged using Fluo-4FF AM for 10 minutes. (B) Electric fields do not induce calcium gradients in cell bodies for 10 minutes or (C) 150 min. (D) EFs do not induce increased [Ca^2+^]_i_ compared with controls, presented as the ratio (F:F0) for 10 minutes or (E) 150 minutes. Scale bar, 20μm.

It is also important to relate cell migration to calcium localization within a cell. There are two potential end points: first, the calcium ions may exit through cathode-facing channels at the speed of the electrical current; second, very little asymmetrically located calcium may remain in the cell because other ions leave the cell to retain a neutral cellular charge. Either of these two possibilities could result in directional cell motility. To test which pathway is preferred by mouse fibroblasts, we used Fluo-4FF AM, an indicator of intracellular calcium, to detect changes in the intracellular calcium content of cells in EFs. The calcium concentrations were detected over short (10 min) and long (150 min) periods. Asymmetrical calcium distribution was not observed in the presence of EFs (Fig 5A–5C), which is consistent with the results of previous studies [33, 37]. In the 10-min experiment, the fluorescence intensity of the indicator increased following a 30-second EF stimulation. However, there was no obvious difference between cells exposed to a current and control cells (Fig 5D and 5E). In the long-term experiment, the fluorescence intensity of the indicator did not increase. These results indicated that the calcium concentration did not change.

Our results demonstrated that cell movement was driven by the flow of calcium ions via inorganic Ca^2+^ channels and SOCs, which were not impacted by BAPTA-AM or by changes in the calcium concentration, consistent with the results of other studies [28, 33]. Therefore, we concluded that the calcium ions passed through the cells rather than being retained by the cells. This conclusion supported the hypothesis that BAPTA-AM did not chelate the influx of calcium ions, and thus did not inhibit cathode-directed cell movement because the influx of calcium ions was not retained by the cells.

### Mouse Fibroblasts Use the RTK-PI3K Pathway in Cathode-Directed Migration

**Fig 6:**
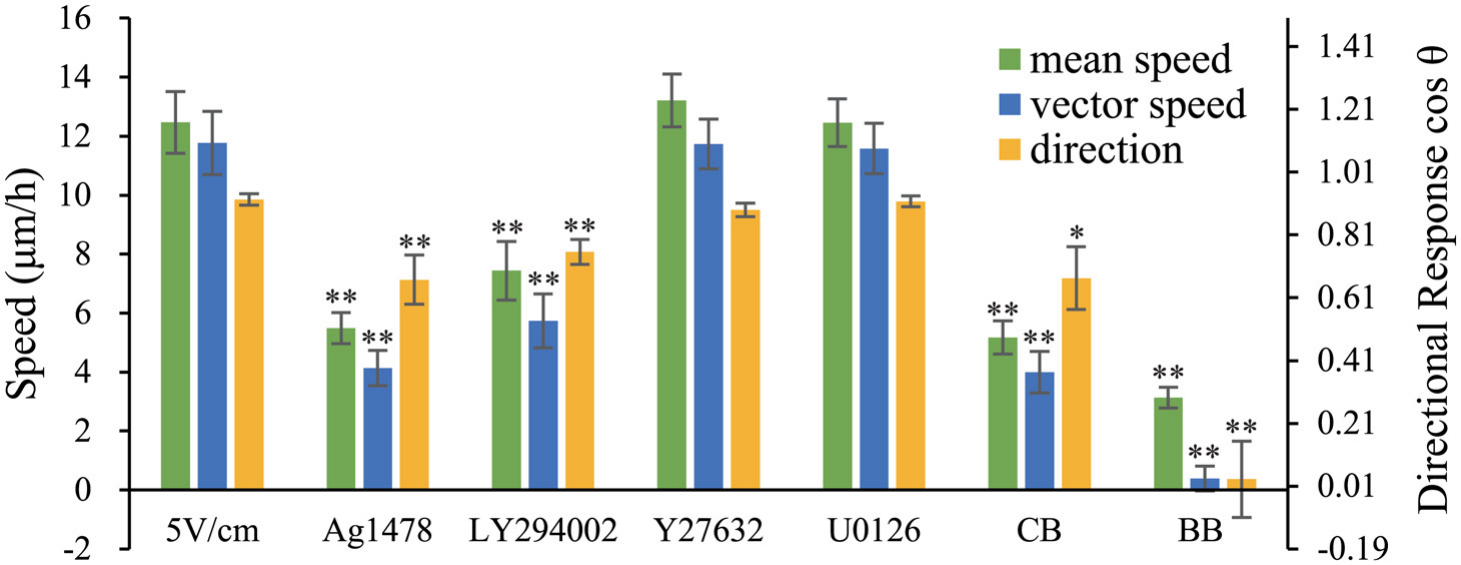
The PI3K Pathway Was Involved in Galvanotaxis. Treatment with the RTK inhibitor AG1478 (10 μM), the PI3K inhibitor LY294002 (30 μM), the actin polymerization inhibitor CB (1 μg/ml) or the myosin-inhibitor BB (25 μM) abolished cathode-oriented cell migration, but treatment with Y27632 (10 μM) or U0126 (10 μM) did not. * (p < 0.05) and ** (p < 0.01) vs EF 5 V/cm control. Error bars indicate standard error.

To test which pathway is involved in the directional movement of mouse fibroblasts, we inhibited the function of receptor tyrosine kinases (RTKs), phosphoinositide 3-kinase (PI3K), mitogen-activated protein kinase (MAPK) and Rho-associated kinase (ROCK), which have been reported to be involved in galvanotaxis and chemotaxis [12, 38], using the inhibitors AG1478(10 μM), LY294002 (30 μM), U0126 (10 μM)and Y27632 (10 μM), respectively. The results showed that AG1478 and LY294002 significantly inhibited cathode-directed cell movement but that Y27632 and U0126 did not (Fig 6 and S6 Movie). In addition, blebbistatin (BB) (25 μM), an inhibitor of myosin activity and cytochalasin B (CB) (1 μg/ml), an inhibitor of actin polymerization, each inhibited cathode-directed cell migration (Fig 6 and S6 Movie), which implied that the assembly of myosin- and actin-containing structures may be the final intracellular process necessary for cell migration. Arguably, RTK recruits PI3K via SH2 domains [39], and the PI3K pathway is responsible for Arp2/3-mediated F-actin polymerization [40]. We concluded that intracellular calcium-ion flow targeted the RTK- PI3K pathway, which mediates cytoskeletal reorganization.

### PC3 Cells Utilized the Same Mechanism as Mouse Fibroblasts

**Fig 7:**
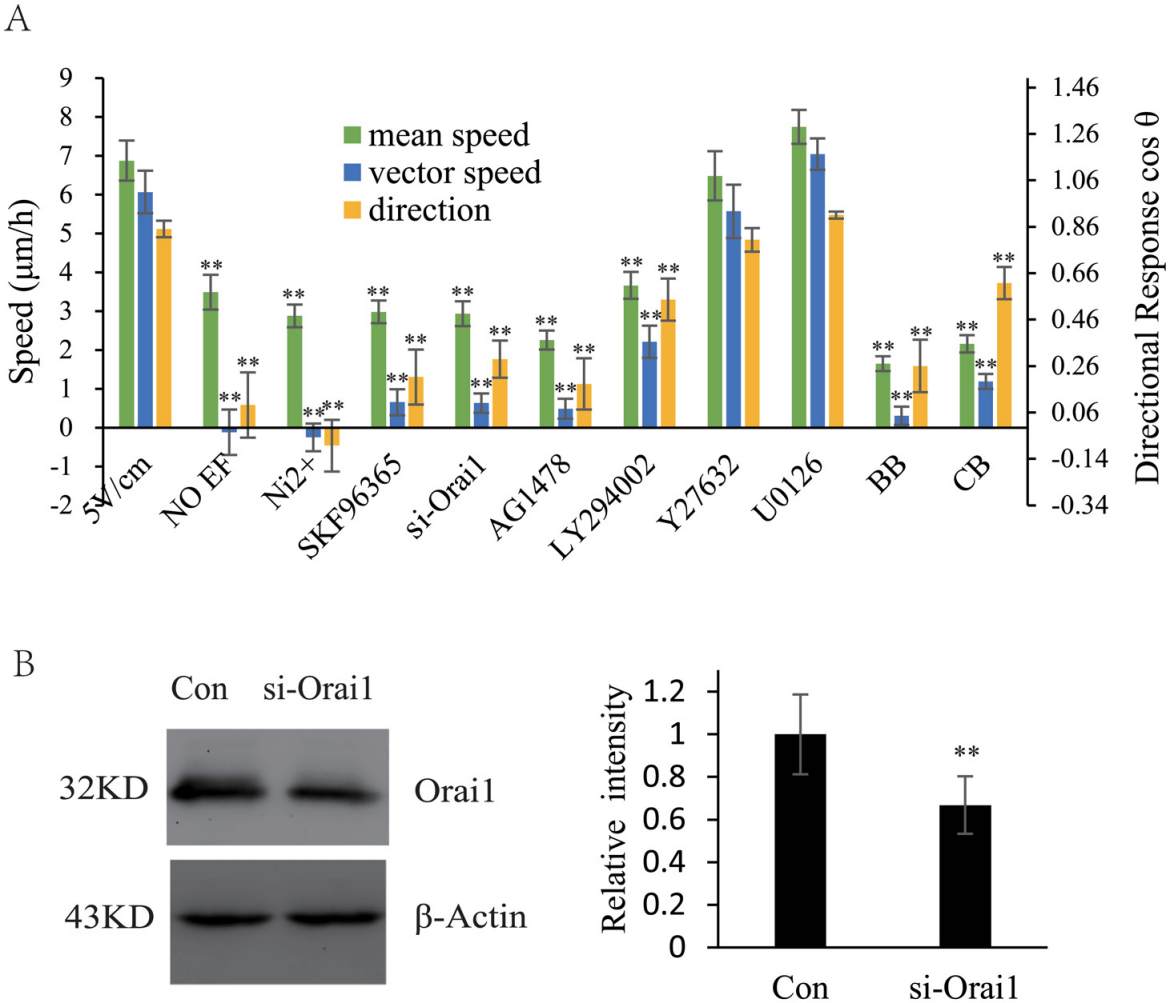
PC3 cells Utilized the Same Galvanotactic Mechanism as Mouse Fibroblasts. (A) Ni^2+^ (300 μM), SKF96365 (10 μM), AG1478 (10 μM), LY294002 (30 μM), Y27632 (10 μM), U0126 (10 μM), BB (25 μM), CB (1 μg/ml) and knocking down Orai1 expression restricted the galvanotaxis of PC3 cells as well as mouse fibroblasts. (B) The efficiency of the siRNA-mediated knockdown was evaluated using western blotting. n = 3. * (p < 0.05) and ** (p < 0.01). Error bars indicate standard error.

To confirm that the cells that migrate toward the cathode employ a universal mechanism, we explored another line of epithelioid cells [41], PC3 cells, which also migrated toward the cathode. We used Ni^2+^ (300 μM), SKF96365 (10 μM), AG1478 (10 μM), LY294002 (30 μM), Y27632 (10 μM), U0126 (10 μM), BB (25 μM) and CB (1 μg/ml) to inhibit the inorganic Ca^2+^ channels, SOCs, the RTK, PI3K, ROCK and MAPK pathways, myosin activity and actin polymerization, respectively, and we obtained results that were the same as those obtained using mouse fibroblasts (Fig 7 and S7 Movie). To demonstrate that SOCs were involved, the level of expression of the calcium release-activated calcium channel protein 1 (Orai1) protein, which constitutes the pore-forming subunits of the SOCs was knocked down using siRNA, and the cathode-oriented migration of the Orai1 knockdown cells was significantly inhibited. These results supported the conclusion that human prostate cancer PC3 cells share the mechanism utilized by mouse fibroblasts to move under EF conditions.

## Discussion

**Fig 8:**
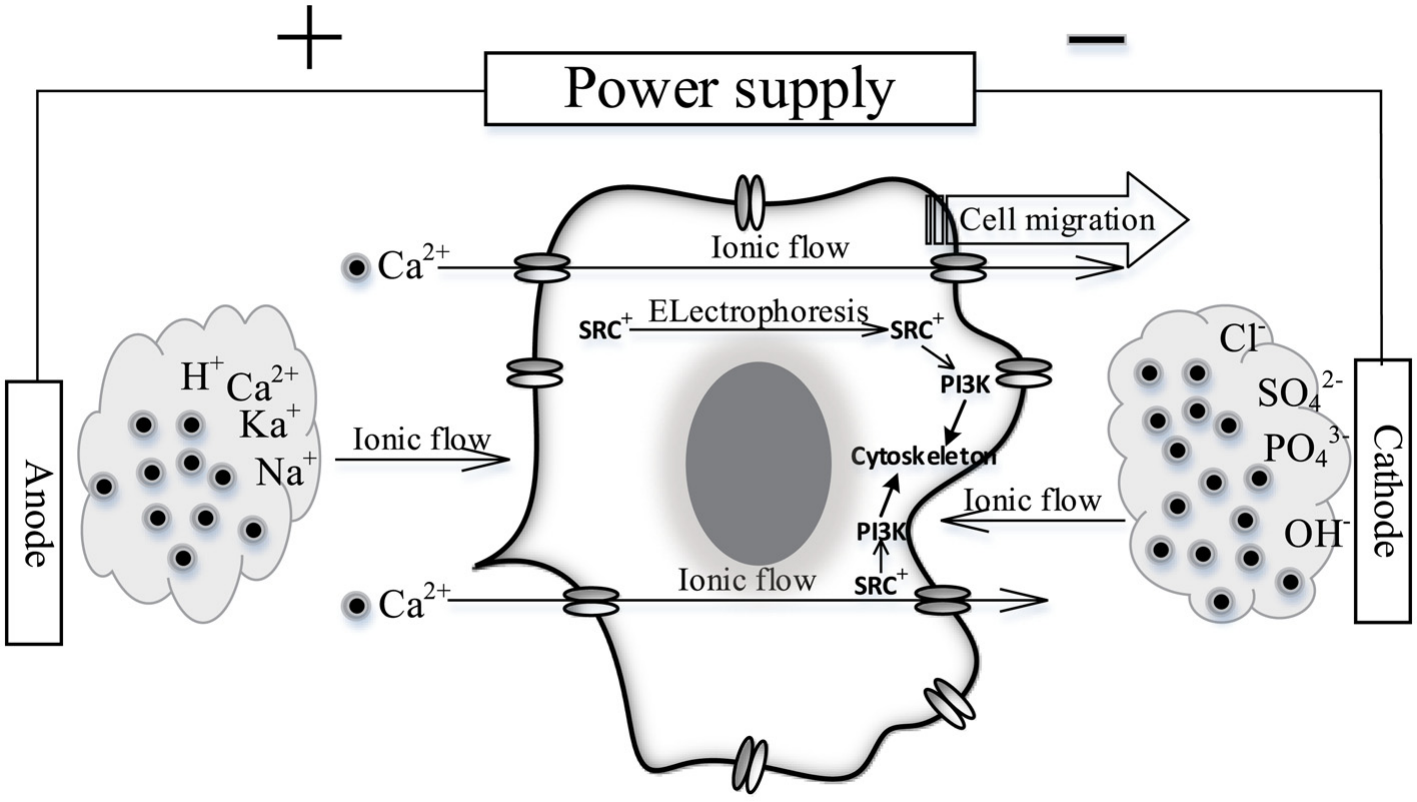
Schematic Illustration of Fibroblast Directional Sensing in EF. In fields of direct current, cations move toward the cathode, and anions move toward the anode. Calcium ions permeate the cell through SOCs at the drift velocity, and this was the primary physical mechanism for directional cell motility and sensation of the electrical field. A positively charged RTK moves to the cathode-facing membrane in an electrophoretic process and recruits the PI3K to that surface; then, the PI3K pathway is responsible for cytoskeletal polymerization and cell migration toward the cathode.

It has been noted that cell migration in an EF does not occur via simple electrophoresis but rather involves complex signal transduction and cytoskeletal remodeling [42]. Thus, we postulated that sensing the calcium-ion flow, which is a component of the EF applied to the medium, would be feasible for cells in vivo. During the embryonic development of some animals and after wounding, many tissues generate electrical potentials through directional ion transport, which arises from spatial variations in the functioning of ion pumps or ion leakage across individual cells or layers of cells [3]. Ion transport through ion channels or within the intercellular space guides cellular movement. Based on the results of our studies, we propose that small intracellular electrical potentials are derived from calcium passing through cells via SOCs and that the cathode-oriented cell migration was induced by the intracellular electrical potentials activating the RTK-PI3K pathway (Fig 8).

The role of calcium in cathode-oriented cell migration is indisputable [30, 32]. However, many hypotheses were disrupted by the finding that the intracellular calcium chelator BAPTA cannot inhibit cell galvanotaxis [28, 33]. Although we suggested that calcium ions permeated such cells through SOCs, this was not a direct observation but rather a speculation. The direction of calcium ion flow within the medium ensured that calcium could enter the cell only through the anode-facing calcium channels, and this mode of entry was very different from that of calcium during chemotaxis [43]. During chemotaxis, the flow of calcium ions must be accompanied by other ions to maintain a neutral cell charge, so that calcium ions can enter and remain within the cell. However, an electrical current flows in a circle. In our galvanotaxis system, calcium ion flow was independent of negative-ion flow and did not cease until it reached the cathode pool, which was separated from the cells by an agar bridge. The calcium electrical current was unaffected by treatment with BAPTA-AM or Fluo-4FF AM.

It should be noted that this study examined only the initial recognition of the EF by the cell, and the processes subsequent to calcium transit through the cell remain unresolved. Calcium transit through the cell from the anode face to the cathode face would appear to lead to asymmetrical cytoplasmic signaling, which may be the role of the RTK-PI3K pathway. Our results indicated that the RTK-PI3K pathway was involved in galvanotaxis, and others have reported Src accumulation and inositol-phospholipid signaling on the cathode faces of cells [12]. However, the basis of the relationship between the flow of calcium ions and RTK-PI3K polarization is unknown. One possible explanation is electrophoresis. The isoelectric point of Src from mice or humans is 7.95 or 7.22, respectively, as calculated by the ProtParam tool [44], which would cause it to be positively charged and to exhibit cathode-oriented movement at pH 7.0 in a direct-current electrical field. Some studies of cell migration found that the calcium level was higher in the rear than in the front of a migrating cell [26]. During chemotaxis, calcium ion influx is activated [43], and PI3K and phospholipase C signaling exhibit asymmetry [45]; thus, calcium ion flow must occur. Whether the localization of these signaling molecules is controlled by the intracellular calcium flow during galvanotaxis remains to be determined. If so, the PI3K pathway can mediate cytoskeletal polymerization and cell migration in the cathode direction.

In addition to a calcium ion-mediated mechanism, other mechanisms may participate in electrical field-mediated cell reorientation. When the calcium ions in the medium were chelated or the calcium channels were blocked, mouse fibroblasts were still able to reorient and to lie perpendicular to the field vector. These results implied that depolymerization occurred within the cytoskeleton facing the two electrodes and that repolymerization occurred within the cytoskeleton perpendicular to the field vector and was not subject to the calcium ion flow (S4 and S5 Movies).

Further research to elucidate how calcium ion flow and electrical potentials regulate intracellular signaling and organelles may identify the under-studied migratory mechanism utilized in directional cell motility in an EF and may reveal new approaches that may be useful in clinical applications.

## Supporting Information

S1 Movie A 5 V/cm electrical field guided fibroblast migration toward the cathode for 21 h. (MP4)

S2 Movie A 5 V/cm electrical field guided PC3 cells toward the cathode for 7 h. (MP4)

S3 Movie The inhibitory effect of EGTA in the cathode or anode culture medium on the galvanotaxis of mouse fibroblasts. (MP4)

S4 Movie The inhibitory effects of Ni^2+^, Gd^3+^, La^3+^ and Sr^2+^ on mouse fibroblast galvanotaxis. (MP4)

S5 Movie The inhibitory effects of 2-APB, SKF96365, Nifedipine and BAPTA-AM on mouse fibroblast galvanotaxis. (MP4)

S6 Movie The inhibitory effects of LY294002, AG1478, Y27632 and U0126 on mouse fibroblast galvanotaxis. (MP4)

S7 Movie A 5 V/cm electrical field guided PC3 cells to the cathode. The effects of AG1478, SKF96365 and Orai1 knockdown on PC3 cell galvanotaxis are illustrated.
(MP4)

S1 Table Raw data of Fig 1D. (XLSX)

S2 Table Raw data of Fig 3B. (XLSX)

S3 Table Raw data of Figs 2, 3, 4 and 6. (XLSX)

S4 Table Raw data of Fig 7. (XLSX)
